# A Diagnostic Chart of Tumors and Pseudo-Tumors

**Published:** 1903-10

**Authors:** 


### A Diagnostic Chart of Tumors and Pseudo-Tumors.

Battle & Co. have just issued a complete and unique chart on the above subject, compiled by Dr. Edward C. Hill from standard works on surgery and pathology. The subject matter is divided into solid neoplasma (subdivided into benign and malignant growths) and true and false cysts. The general characteristics of each division are given, and their twenty-four classes, embracing over 100 varieties, are compared critically in columns under the following headings: Tissue, Topography, Number, Size, Conformation, Color, Consistence, Mobility, Sensibility, Surrounding Tissues, Occurrence, History of Growth, and Miscellaneous Points. Features of special differential value are emphasized by the use of italics. This chart shows almost at a glance for ready comparison all that could be learned in a diagnostic way from the perusal of hundreds of pages of ordinary text. It stands, indeed, to such books as an atlas does to a gazetteer. This very convenient and valuable compendium is at the command gratis of any and every practitioner of medicine, who will take the trouble of writing a postal card to Battle & Co., 2001 Locust Street, St. Louis.

